# Primary osteogenic sarcoma of the breast

**DOI:** 10.1186/1477-7819-4-90

**Published:** 2006-12-11

**Authors:** Temidayo O Ogundiran, Samuel A Ademola, Odunayo M Oluwatosin, Effiong E Akang, Clement A Adebamowo

**Affiliations:** 1 Division of Oncology, Department of Surgery, University College Hospital, Ibadan, Nigeria; 2 Division of Plastic and Reconstructive Surgery, Department of Surgery, University, College Hospital, Ibadan, Nigeria; 3 Department of Pathology, University College Hospital, Ibadan, Nigeria

## Abstract

**Background:**

Primary extra-osseous osteogenic sarcomas have been reported in many tissues of the body but their occurrence in the breast is extremely rare. It can arise as a result of osseous metaplasia in a pre-existing benign or malignant neoplasm of the breast or as non-phylloides sarcoma from the soft tissue of a previously normal breast.

**Case presentation:**

A 40 year-old Nigerian woman was clinically diagnosed to have carcinoma of the left breast. The histology report of core-needle biopsy of the mass showed a malignant neoplasm comprising islands of chondroblastic and osteoblastic stromal cells. This report changed the diagnosis from carcinoma to osteogenic sarcoma of the breast. She had a left modified radical mastectomy, however there was significant post surgery skin deficit. A *latissimus dorsi *musculocutaneous flap was used to cover the anterior chest wall defect. Sections from the mastectomy specimen confirmed the diagnosis of osteogenic sarcoma. She died six months after mastectomy.

**Conclusion:**

A diagnosis of osteogenic sarcoma of the breast was made based on histology report and after excluding an osteogenic sarcoma arising from underlying ribs and sternum. This is the second documented case of primary osteogenic sarcoma of the breast coming from Nigeria

## Background

Breast cancer is the commonest cancer that afflicts females worldwide. In Cancer Statistics 2005, breast cancer remains the leading cancer among American women with an estimate of 32% excluding skin cancers [1]. Of all the cancers of the breast, carcinoma forms the bulk while breast sarcomas are negligible [2,3]. Extra-skeletal osteosarcoma has been documented in many tissues of the body including the thyroid gland, kidney, bladder, colon, heart, testis, penis, gall bladder and the cerebellum [4-10]. When it occurs in the breast, it originates either from normal breast tissue *de novo*, or as metaplastic differentiation of a primary benign or malignant breast lesion. Osteogenic sarcomas of the breast either arising primarily in the breast or as secondary deposits from primary bone sarcomas occur in very rare cases.

Almost every previous reference to this entity in literature is in form of single case reports. In almost all cases, the patients had been diagnosed clinically as having breast carcinoma and the correct tissue diagnosis was established by histology [11,12]. The largest collection of primary breast osteogenic sarcomas found on Pubmed search from 1967 to date was a clinico-pathological analysis of 50 cases seen over a 38-year period and reported by Silver and Tavassoli in 1998 [13]. This paper reports the case of a young woman who presented with recurrent left breast lump which was clinically diagnosed as carcinoma but turned out to be osteogenic sarcoma arising from the breast.

## Case presentation

A 40 year-old Nigerian housewife was seen at the oncology clinic of the University College Hospital (UCH) Ibadan, Nigeria in June 2002 with a 1 year 8 months history of painful left breast lump which had been previously excised in another hospital but recurred 8 months before presentation at UCH. There was no information about histological diagnosis of the excised breast lesion from the first hospital. There were no systemic symptoms. She was Para 7^+1 ^and had no family history of breast or ovarian cancer. Physical examination revealed globular enlargement of the left breast measuring 20 cm × 18 cm. The mass occupied the whole breast, was warm, multinodular and fixed to the *pectoralis *fascia. The ipsilateral axillary lymph nodes were enlarged, but examination of the other systems was normal. A clinical diagnosis of locally advanced cancer of the left breast was made.

**Figure 1:**
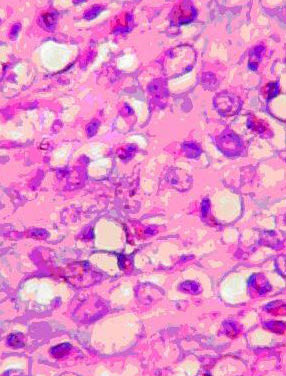
Photomicrograph from breast neoplasm displaying cartilaginous differentiation of malignant stromal elements (Hematoxylin-eosin, × 440).

**Figure 2:**
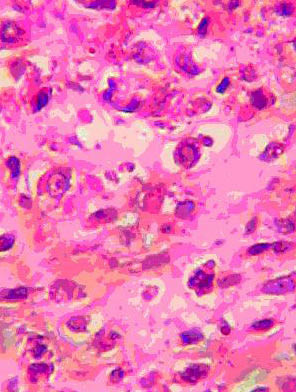
Photomicrograph from tumor displaying osteoid deposition by the malignant stromal cells (Hematoxylin-eosin, × 440).

Plain radiograph of the chest and abdominal ultrasound scan were normal. A core-needle biopsy of the mass was done and histology showed a malignant neoplasm comprising islands of chondroblastic and osteoblastic stromal cells, with no normal breast tissue seen. A diagnosis of osteogenic sarcoma was made. The patient had a left modified radical mastectomy and *latissimus dorsi *musculocutaneous flap to cover an anterior chest wall defect. The mastectomy specimen weighed 350 g. Cut sections revealed areas of cystic degeneration and necrosis, with focal areas that were firm with a cartilaginous consistency. Conventional representative sections were obtained from each of the four breast quadrants, areola region, resection margins and axillary lymph nodes. Microscopic examination of the sections showed a malignant breast neoplasm displaying fibrosarcomatous, chondrosarcomatous (Figure 1) as well as osteosarcomatous (Figure 2) differentiation. There was metastasis to one of the lymph nodes. She was scheduled for radiotherapy to the chest wall but she defaulted. Contact tracing revealed that she died about 6 months after mastectomy.

## Discussion

Osteogenic sarcoma of the breast tissue can arise from a pre-existing benign or malignant neoplasm of the breast or may arise from previously normal breast tissue as non-phylloides sarcoma. It is known to differentiate from the connective tissue elements of fibroadenomas and has been reported following intraductal papilloma [3,14]. Breast osteosarcoma can also arise as an osseous metaplasia of a primary carcinoma of the breast and as a whole or partial metaplastic replacement of phylloides tumor stroma [15,16]. In its pure form and in exceptional cases, osteogenic sarcoma can arise from the soft tissues of a previously normal breast [17]. To diagnose primary breast osteosarcoma, an osteogenic sarcoma arising from the underlying chest wall bony cage and infiltrating the breast tissue must be excluded. From findings at surgery and from the pathology report, our patient did not have gross or microscopic chest wall osteosarcoma extending to the breast. The absence of demonstrable metaplastic transformation of a pre-existing fibroadenoma or phylloides tumor at histology among others, suggests that the case presented could be an example of breast sarcoma arising from previously normal breast tissue.

Almost all patients with osteogenic sarcoma present like those with other benign or malignant breast lesions. Clinically, the breast lump is of varying consistency and may be rapidly growing. At the time of presentation, most patients have developed metastasis in different parts of the body including the chest and bones, though this was not the case with our patient [18]. The two patients from our centre – this case and the earlier reported one [11] – presented for definitive treatment about one year after noticing breast lumps and when the tumors were already locally advanced. This is typical of breast cancer patients about 80 percent of who present late to our oncology clinic [19]. Mammographic findings vary. Most present as large masses with relatively well-defined margins and lobulated borders, often containing coarse or dense calcifications, which are sometimes similar to fibroadenomas [20]. Where available intense focal intake of 99mTc-diphosphonate, a specific radionuclide marker for osteoid tumoral tissue, in a soft-tissue tumor is strongly suggestive of bone-forming neoplasms. This may be useful in the radiological diagnosis of breast osteosarcoma [20-22]. Serum alkaline phosphatase is known to be elevated in patients with osteoid-forming neoplasms [20]. Though non-specific, these tests may be useful adjuncts in the diagnosis of osteogenic sarcoma of the breast. Definitive diagnosis hinges on exclusion of an osteogenic sarcoma arising from underlying ribs and sternum, and a demonstration of osteosarcomatous matrix by histology. Where facilities are available, immunohistochemical demonstration of vimentin with absence of epithelial, neural, muscular and other markers suggest the diagnosis of osteogenic sarcoma. This may be confirmed by ultrastructural examination of the osteoid-like areas, which will reveal collagen fibers enmeshed with crystalline material confirming the presence of osteoid [23].

The rarity of this condition precludes any one institution from gathering enough cases from which a definitive assessment of the effectiveness of treatment options can be made or clinical trials conducted. It follows however, that management of sarcomas elsewhere should generally apply in the management of this disease entity. It is debatable whether modified radical mastectomy should be done instead of simple mastectomy with axillary node dissection where there is clinical evidence of lymph node involvement. Adequate surgical excision should ensure tumor free margins and be supplemented by intraoperative evaluation of lymph nodes. Osteosarcomas are aggressive tumors with a propensity for blood-borne rather than lymphatic spread [13]. Because of high risk of recurrence, chest wall irradiation and regular follow-up for early detection of loco-regional recurrence is indicated. The role of combination chemotherapy is uncertain but it may be considered in the presence of systemic metastasis. However, these have not been proven to offer additional benefits.

Because these tumors tend to attain large sizes and the role of neoadjuvant chemotherapy to down-stage them is unclear, patients occasionally require chest wall reconstruction. Myocutaneous flaps are preferred because they can tolerate radiotherapy with minimal risk of tissue loss [24-27]. *Latissimus dorsi *musculocutaneous flap was used for our patient because of the ease of raising it, its reliability and cosmetic acceptability of the scar at the donor site, which is hidden at the back of the patient where it is covered with regular clothing. The major disadvantage of the flap is that patient may have to be repositioned during the course of surgery to ensure improved access.

## Conclusion

We report here another case of osteosarcoma of the breast where definitive diagnosis was made based on histology report and after excluding an osteogenic sarcoma arising from underlying ribs and sternum. To date, adequate surgical excision is the best form of treatment that offers the best outlook for patients. Its rarity, however, should not prevent oncologists from following up on the literature of the disease to keep abreast of new cases and possibly new developments.

## Competing interests

The author(s) declare that they have no competing interests.

## Authors' contributions

**TOO**, **SAA **and **OMO **took part in the care of the patient. **EEA **examined surgical specimen and took photomicrographs of the slides. **TOO **initiated and co-wrote the paper with **SAA**, **CAA **and **EEA**. All authors read and approved the final manuscript.
